# CDT1 is the major functional regulatory subunit of the pre‐replication complex in zygotes

**DOI:** 10.1111/cpr.13377

**Published:** 2022-12-07

**Authors:** Chao Li, Yong‐Peng Tan, Xue‐Shan Ma, Zhen‐Bo Wang, Tie‐Gang Meng, Qing‐Yuan Sun

**Affiliations:** ^1^ State Key Laboratory of Stem Cell and Reproductive Biology Institute of Zoology, Chinese Academy of Sciences Beijing China; ^2^ University of Chinese Academy of Sciences Beijing China; ^3^ Fertility Preservation Lab, Guangdong‐Hong Kong Metabolism & Reproduction Joint Laboratory Reproductive Medicine Center, Guangdong Second Provincial General Hospital Guangzhou China; ^4^ Reproductive Genetics Department The Affiliated Tai'an City Central Hospital of Qingdao University Taian China

## Abstract

Pre‐replication complex (pre‐RC) is critical for DNA replication initiation. CDT1 and MCM2 are the subunits of pre‐RC, and proper regulation of CDT1 and MCM2 are necessary for DNA replication and cell proliferation. The present study aimed to explore the role of CDT1 and MCM2 in oocyte meiotic maturation and early embryonic development. The depletion and overexpression of *Cdt1* and *Mcm2* in oocyte and zygote were achieved by microinjecting specific siRNA and mRNA to explored their functions in oocyte meiotic maturation and embryonic development. Then, we examined the effect of CDT1 and MCM2 on other signal pathways by immunostaining the expression of related maker genes. We showed that neither depletion nor overexpression of *Cdt1* affected oocyte meiotic progressions. The CDT1 was degraded in S phase and remained at a low level in G2 phase of zygote. Exogenous expression of *Cdt1* in G2 phase led to embryo attest at zygote stage. Mechanistically, CDT1 overexpression induced DNA re‐replication and thus DNA damage check‐point activation. Protein abundance of MCM2 was stable throughout the cell cycle, and embryos with overexpressed MCM2 could develop to blastocysts normally. Overexpression or depletion of *Mcm2* also had no effect on oocyte meiotic maturation. Our results indicate that pre‐RC subunits CDT1 and MCM2 are not involved in oocyte meiotic maturation. In zygote, CDT1 but not MCM2 is the major regulator of DNA replication in a cell cycle dependent manner. Furthermore, its' degradation is essential for zygotes to prevent from DNA re‐replication in G2 stage.

## Graphical abstract

Pre‐replication complex (pre‐RC) is formed in G1 phase during which CDT1 is localized in nucleus. When zygote enters S phase, CDT1 is degraded and stays at a low level in G2 phase. However, the expression of exogenous *Cdt1* mRNA in G2 phase of fertilized egg, mimicking events in which CDT1 degradation is disrupted, pre‐RC reassembles and leads to DNA re‐replication, and thus DNA damage check point activation, which results in embryo arrest at G2/M phase.

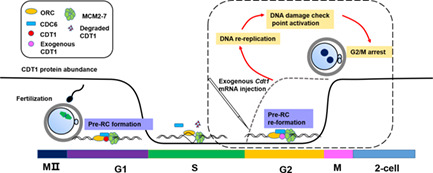

## 1 INTRODUCTION

DNA replication is an essential event in cell division. During mitotic S phase, the cells initiate DNA replication, doubling the number of DNA, and after which, chromosomes are evenly distributed to two daughter cells in M phase. The initiation of DNA replication is tightly regulated, and binding of pre‐replication complex (pre‐RC) to DNA is required for DNA replication in S phase. Pre‐RC composes of numerous subunits, including origin recognition complex (ORC), CDT1, CDC6 and MCM complex. The formation of pre‐RC is a sequential assembling process in which the ORC first binds directly to the DNA, CDC6 and CDT1 are assembled sequentially onto the ORC, and then the MCM complex is recruited.^1^ When cell enters S phase, DNA replication is initiated, CDT1 and CDC6 are released from pre‐RC, leaving MCM2‐7 unwinds DNA through interaction with DNA helicase.^2^, ^3^

CDT1, as a factor in recruiting MCM, is tightly regulated during the cell cycle. *Cdt1* is highly expressed in G1 and G2 phases; when cell enters S phase, CDT1 is ubiquitylated by CRL4^Cdt2^ or CRL1^Skp2^, or binds with geminin to prevent pre‐RC reassembly.^4^, ^5^, ^6^, ^7^, ^8^ Correspondingly, overexpression of *Cdt1* results in re‐replication and genome instability.^9^, ^10^, ^11^ In addition to its role in forming pre‐RC complex, Varma *et al*. find that deletion of CDT1 in G2 phase arrests cell in late prometaphase, for that CDT1 binds with HEC1 is required for stable kinetochore–microtubule attachment.^12^ In meiosis, germ cell divides twice while replicates once. The germinal vesicle (GV) stage oocytes are arrested at prophase of the first meiosis, and they have already completed DNA replication, giving us a good model to investigate the roles of CDT1 in kinetochore–microtubule attachment in meiotic oocyte. In addition, whether CDT1 degradation is essential for embryonic development is unclear.

We recently showed that CDC6, a subunit of pre‐RC, regulates not only the initial replication of DNA, but also the G2/M transition and metaphase‐to‐anaphase transition during oocyte meiosis.^13^ In addition to CDC6, we are interested in the function of other subunits of pre‐RC, such as CDT1 and MCM complexes. In order to explore the potential roles of CDT1 and MCM2 in oocyte meiotic maturation and embryonic development, we knock down *Cdt1* or *Mcm2* expression by microinjecting *Cdt1* and *Mcm2* specific siRNA in GV oocytes, respectively, and exogenously expressed CDT1 or MCM2 by microinjecting *Cdt1* mRNA or *Mcm2* mRNA in GV oocytes and zygotes. We found no significant function of CDT1 an MCM2 in oocyte meiotic maturation in vitro. But the overexpression of *Cdt1* in G2 phase results in embryo arrest at zygote stage, which is due to DNA re‐replication and DNA damage pathway activation. Thus, our data deepens our understanding of pre‐RC function in oocyte meiotic maturation and early embryonic development.

## 2 MATERIALS AND METHODS

### 2.1 Oocyte and zygote collection and culture

Care and handing of ICR mice were conducted in accordance with policies promulgated by the Animal Ethics Committee of Guangdong Second Provincial General Hospital. The GV oocytes were collected from ovaries in M2 medium (Sigma‐Aldrich) supplemented with 1 μM milrinone. To collect zygotes, ICR mice received intraperitoneal injection of 10 IU PMSG, followed by 10 IU hCG injection after 46 h, then mated with male mouse, and vaginal plud was checked on the morning of the second day. The zygotes were collected from oviducts after 18 h of hCG injection, and cumulus cells were removed by treatment with 0.5 mg/ml hyaluronidase (Sigma, St. Louis, MO, USA) in the M2 medium.

For IVF, spermatozoa were collected from epididymis of male ICR mice and pre‐incubated in HTF medium for 1 h at 37°C, 5% CO_2_. The MII oocytes were inseminated with capacitated spermatozoa for 4 h, then fertilized eggs were washed and cultured in KSOM medium.

### 2.2 Quantitative real time‐PCR

A total of 20 oocytes or embryos were lysed by cell lysis buffer for reverse transcription reactions (Thermo, K1682). We used SYBR green (Vazyme, Q111‐02) for Quantitative real time‐PCR (qRT‐PCR). Primers for *Cdt1* were 5′‐CTCCACAATCGCTCTGAGACTG‐3′ (forward) and 5′‐GGAAGCGATACGACGTGGGATA‐3′ (reverse), primers for *Mcm2* were 5′‐CCGTTCCAAGGATGCCATTCTC‐3′ (forward) and 5′‐TGGAAAGCCGTTGGCGGTGTTA‐3′ (reverse), primers for *Mcm5* were 5′‐GGAGGCATTGAGACTGTTCCAG‐3′ (forward) and 5′‐AGACACCTGAGAGCCAATGGCA‐3′ (reverse), primers for *Mcm7* were 5′‐CAGCTCCTATCTTACATCGACCG‐3′ (forward) and 5′‐GTCAGTTCTCCACTCACAGAGTC‐3′ (reverse), the reference gene was GAPDH, and primers were 5′‐AGGTCGGTGTGAACGGATTTG‐3′ (forward) and 5′‐TGTAGACCATGTAGTTGAGGTCA‐3′ (reverse).

### 2.3 BrdU labelling for DNA replication

The zygotes were cultured in KSOM medium with 100 μm BrdU (858,811; Sigma‐Aldrich), then were fixed with PFA (4% paraformaldehyde in PBS) for 15 min, permeated with PBST (0.5% Triton X‐100 in PBS) for 20 min, treated with 4 N HCl for 10 min, and neutralized with 100 Mm Tris–HCl (pH 8.5) for 10 min at room temperature. Subsequently, the zygotes were blocked in BSA for 1 h at room temperature, then incubated with anti‐BrdU antibody (1:1000;B8434; Sigma‐Aldrich) at 4°C overnight, washed with PBST every 10 min for four times, and finally incubated with secondary Alexa Fluor 488–conjugated antibody (1:1000; A11001; Life Technologies, Shanghai, China).

### 2.4 Western blot

Oocytes and embryos were collected in protein lysis buffer followed by adding protein loading buffer and heated at 95°C for 10 min. SDS–PAGE and immunoblots were performed according to standard procedures. The following antibodies were used: anti‐CDT1 polyclonal body (Abcam, ab202067), anti‐MCM2 polyclonal body (Cell Signaling Technology, 3619 T) and mouse polyclonal anti‐α‐tubulin antibody (Sungene, KM9007T).

### 2.5 Immunofluorescence and confocal imaging

Oocytes and embryos were fixed for 25 min in 4% paraformaldehyde in PBS, permeabilized for 25 min in 0.5% Triton X‐100 in PBS, blocked in PBS containing 1 mg/ml BSA (1% BSA) for 1 h at room temperature. After blocking, they were incubated with rabbit polyclonal anti‐CDT1 antibody (1:200) and rabbit polyclonal anti‐MCM2 antibody (1:200) at 4°C overnight, respectively.

After washing four times in PBS with 0.1% Tween and 0.01% Triton X‐100 (wash buffer), oocytes or embryos were incubated with FITC‐conjugated goat anti‐rabbit IgG (1:200) at room temperature for 1 h. then they were washed four times in wash buffer and stained with DAPI for 15 min at room temperature. Finally, the samples were mounted on glass slides and visualized with confocal microscopy.

### 2.6 Microinjection of mRNA or siRNA

mRNA or siRNA was transferred into cytoplasm of cells by microinjection. The concentration of siRNA used was 10 μM, *Cdt1* (5′‐GCUCUGAGACUGUGACCUUTT‐3′), *Mcm2* (5′‐GCAUCAGUGAUAUGUGCAATT‐3′), *Mcm5* (5′‐GCAUUGAGAAGCAACUCAATT‐3′) and *Mcm7* (5′‐CCAGGAAUGAAGAUUCAATT‐3′) were used to deplete the expression of corresponding genes. The T7 message machine (Invitrogen, AM1344) and poly (A) tailing kit (Invitrogen, AM1350) were used for transcribing mRNA in vitro. After injection, the oocytes were arrested at the GV stage in M2 medium with milrinone for 24 h to deplete the corresponding gene expression. Then oocytes were released from milrinone through washing with milrinone‐free medium.

### 2.7 Statistical analysis

Data from at least three independent experiments were analysed by GraphPad Prism 10, and presented as mean ± SEM. The *p* < 0.05 was considered as statistically significant in evaluating by student's *t*‐test.

## 3 RESULTS

### 3.1 The expression and subcellular localization of CDT1 and MCM2 in oocyte and embryo

**FIGURE 1:**
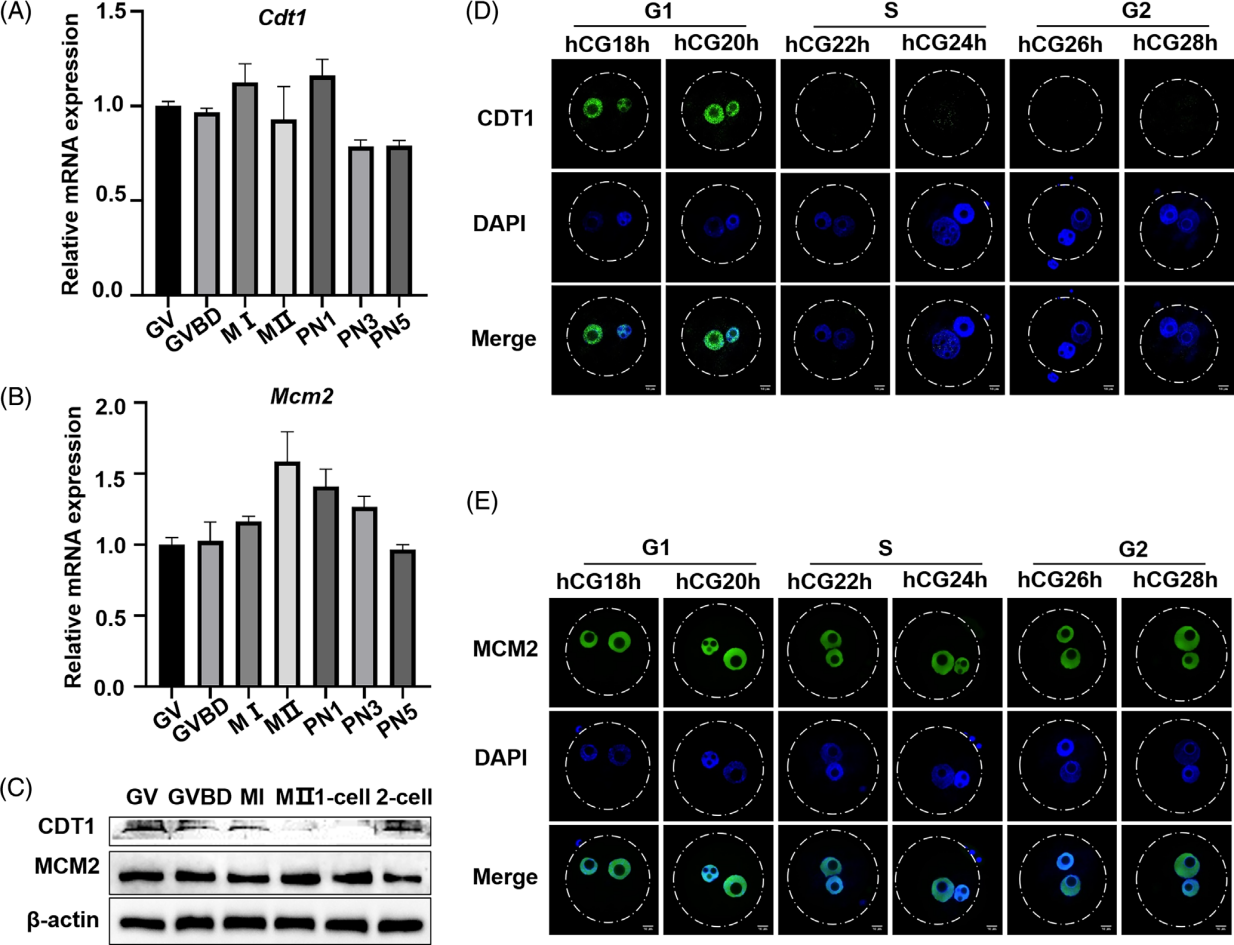
The expression and subcellular localization of CDT1 and MCM2 in oocyte and zygote. (A, B) Oocytes were collected at 0, 3, 8 and 12 h in culture, corresponding to the GV, GVBD, MI and MII stages, respectively. Zygotes were collected at hCG18, 22 and 28 h, corresponding to PN1, PN3 and PN5 stages, respectively. The relative *Cdt1* and *Mcm2* mRNA levels were measured by qRT‐PCR. (C) Protein levels of CDT1 and MCM2 during oocyte meiotic maturation and early embryonic development were identified by Western blot. (D, E) Representative immunofluorescence images of CDT1 and MCM2 expression at distinct stages of fertilized egg development. Scale bar: 10 μm

We first examined the expression patterns of *Cdt1* and *Mcm2* in oocytes and embryos. Quantitative real‐time PCR (qRT‐PCR) revealed the mRNA levels of *Cdt1* and *Mcm2* during different stages of oocyte maturation and embryonic development (Figure 1A,B). Inconsistent with the persistent expression of MCM2 from oocyte to two‐cell embryo, CDT1 protein level almost disappeared during the zygote period (Figure 1C). Because CDT1 was critical for pre‐RC formation before S phase, we explored the expression patterns of CDT1 at different stages of zygotes by immunofluorescence staining. As shown in Figure 1D, CDT1 was localized in the nucleus until S stage, and subsequently disappeared lasting to pronuclear fusion. As for MCM2, it was localized in the pronuclei from PN1 to PN5 stages (Figure 1E).

### 3.2 CDT1 is dispensable for oocyte meiotic maturation

**FIGURE 2:**
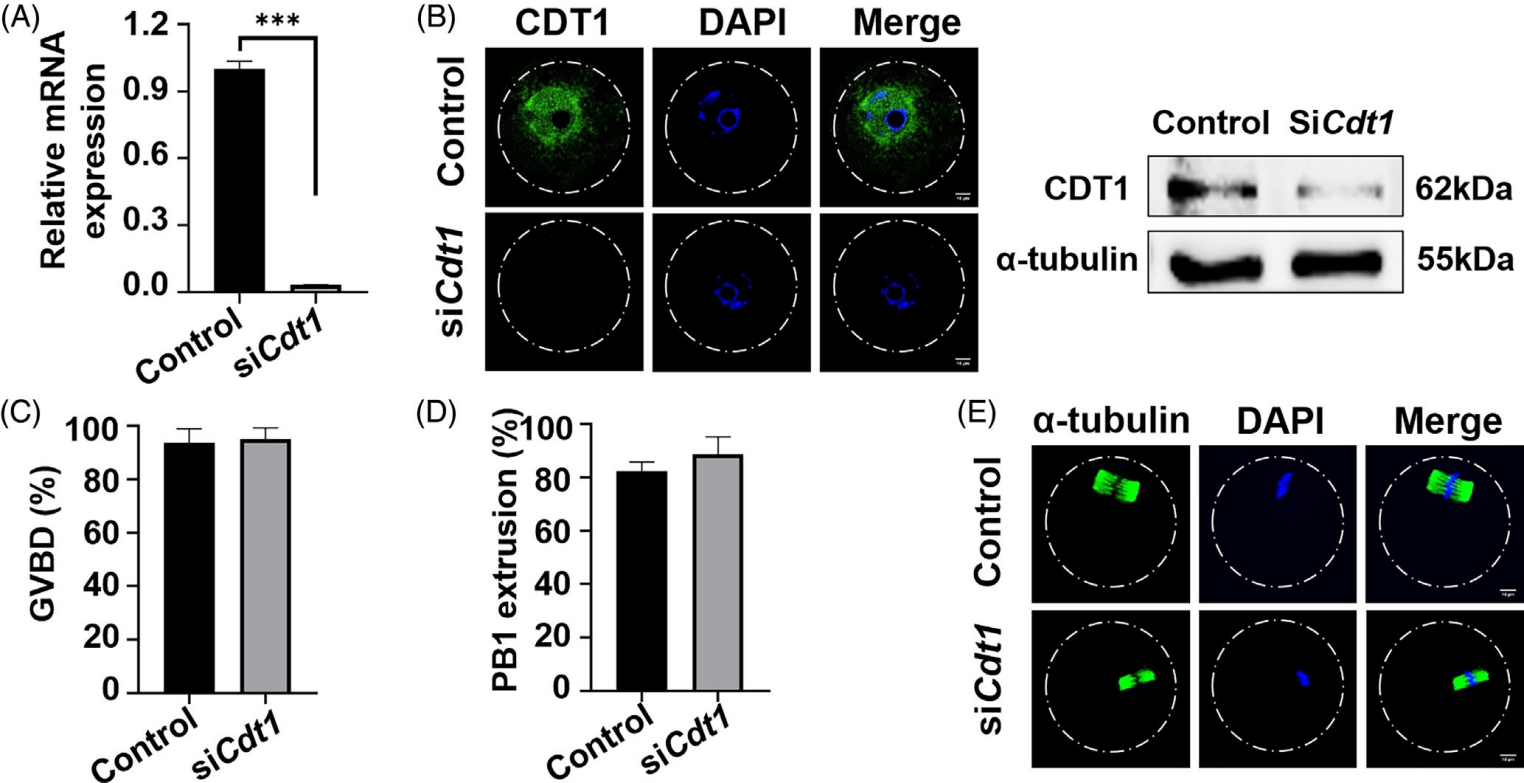
CDT1 is dispensable for oocyte meiotic maturation. (A) Relative mRNA level of *Cdt1* in the oocytes injected with *Cdt1* siRNA or control siRNA. The oocytes were arrested at GV stages for 24 h (***p < 0.001). (B) Representative immunofluorescence and Western blot images of oocytes injected with *Cdt1* siRNA or control siRNA. The molecular mass of CDT1 is 62 kDa, and that of α‐tubulin is 55 kDa. The oocytes were arrested at GV stages for 24 h and collected. For immunofluorescence，a total of 21 oocytes in control group and 19 oocytes in siRNA group were used from three replicates. In Western blot, each sample contained 120 oocytes. (C) The GVBD rates of oocytes injected with *Cdt1* siRNA or control siRNA. (D) The PBE rates of oocytes injected with *Cdt1* siRNA or control siRNA. A total of 91 oocytes in control group and 83 oocytes in siRNA group were used from three replicates. (E) The spindles of MII of oocytes injected with *Cdt1* siRNA or control siRNA. Oocytes were cultured for 24 h in M2 medium with milrinone, and then moved to milrinone‐free medium for culture, followed by immunofluorescent staining. Scale bar: 10 μm

Given that depletion of CDT1 in Hela cells leads to cell arrest due to abnormal kinetochore–microtubule attachment,^12^ we wanted to know whether CDT1 has a similar role in meiosis, so we knocked down *Cdt1* expression by microinjecting its specific siRNA. qRT‐PCR revealed that *Cdt1* mRNA was significantly reduced(Figure 2A). Similarly, the protein abundance of CDT1 was also decreased in *Cdt1* knock down group (Figure 2B).

Next, we asked whether CDT1 changes affected GVBD or PBE. *Cdt1* expression depletion did not affect the proportion of GVBD compared to the control group (Figure 2C). PBE was not affected by *Cdt1* expression knock down, either (Figure 2D). In addition, the MII spindles of CDT1 depleted oocytes were normal compared to control group (Figure 2E). Thus, our data suggest that depletion of *Cdt1* expression does not affect oocyte meiotic maturation.

### 3.3 Exogenous expression of *Cdt1* does not induce DNA re‐replication and has no effect on meiotic maturation of oocyte

**FIGURE 3:**
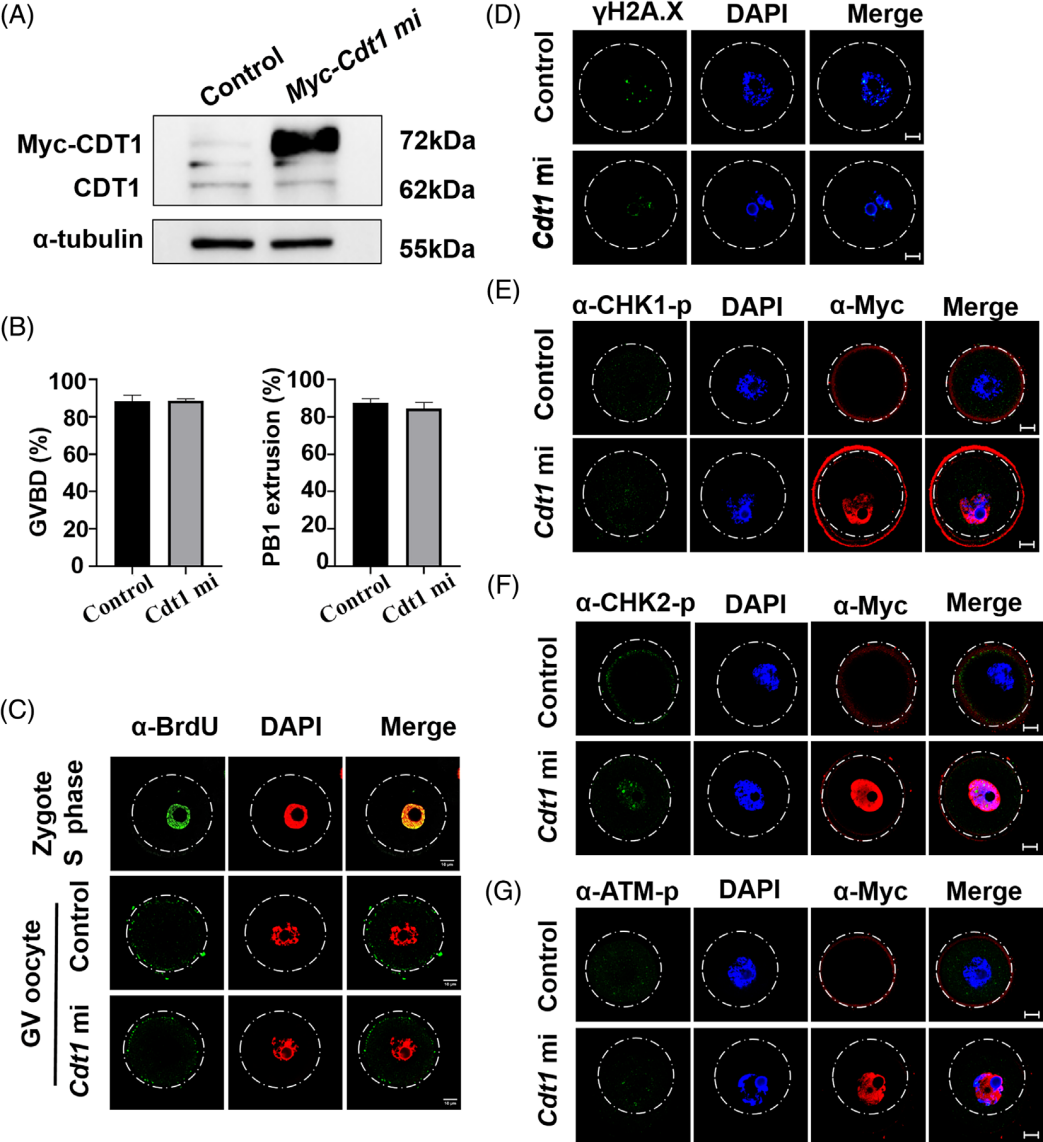
Exogenous expression of *Cdt1* has no effect on oocyte meiotic maturation. (A) Western blot of GV oocytes microinjected with *Myc*‐*Cdt1* mRNA. The molecular mass of CDT1 is 62 kDa, and that of Myc‐CDT1 is 72 kDa and α‐tubulin is 55 kDa. Each sample contained 120 oocytes. (B) The GV oocytes microinjected with *Cdt1* mRNA showed normal GVBD and PBE rates. A total of 100 oocytes in control group and 97 oocytes in mRNA group were used from three replicates. (C) Representative immunofluorescence images of BrdU staining in oocytes^exo‐Cdt1^. (D) Representative immunofluorescence images of γH2A.X staining in oocytes^exo‐Cdt1^. (E) Representative immunofluorescence images of α‐CHK1 phosphorylation staining in oocytes^exo‐Cdt1^. (F) Representative immunofluorescence images of α‐CHK2 phosphorylation staining in oocytes^exo‐Cdt1^. (G) Representative immunofluorescence images of α‐ATM phosphorylation staining in oocytes^exo‐Cdt1^. Scale bar: 10 μm

It has been reported that overexpression of *Cdt1* during S phase leads to DNA re‐replication.^9^ The GV oocytes are arrested at meiotic prophase, during which DNA has already completed replication. Then we ask whether overexpressed CDT1 in GV oocytes could lead to DNA re‐replication. We constructed *Cdt1* plasmid with Myc tag before its N terminal. Then mRNA encoding Myc‐CDT1 was injected into the GV oocytes (Figure 3A), but found that overexpression of CDT1 did not affect GVBD and PBE (Figure 3B). Next, we tested whether DNA could re‐replicate with BrdU staining，but we did not find new DNA synthesis (Figure 3C). At the same time, exogenous expression of *Cdt1* did not result in DNA damage, for that immunofluorescence intensity of γH2A.X was equal between the two groups (Figure 3D). Moreover, other signals related to DNA repair, such as CHK1, CHK2 and phosphorylated ATM were also not affected by exogenously expressed CDT1 (Figure 3E–G). These results suggest that overexpression of CDT1 does not initiate DNA re‐replication in prophase of oocyte.

### 3.4 Exogenous expression of *Cdt1* leads to embryonic development arrest

**FIGURE 4:**
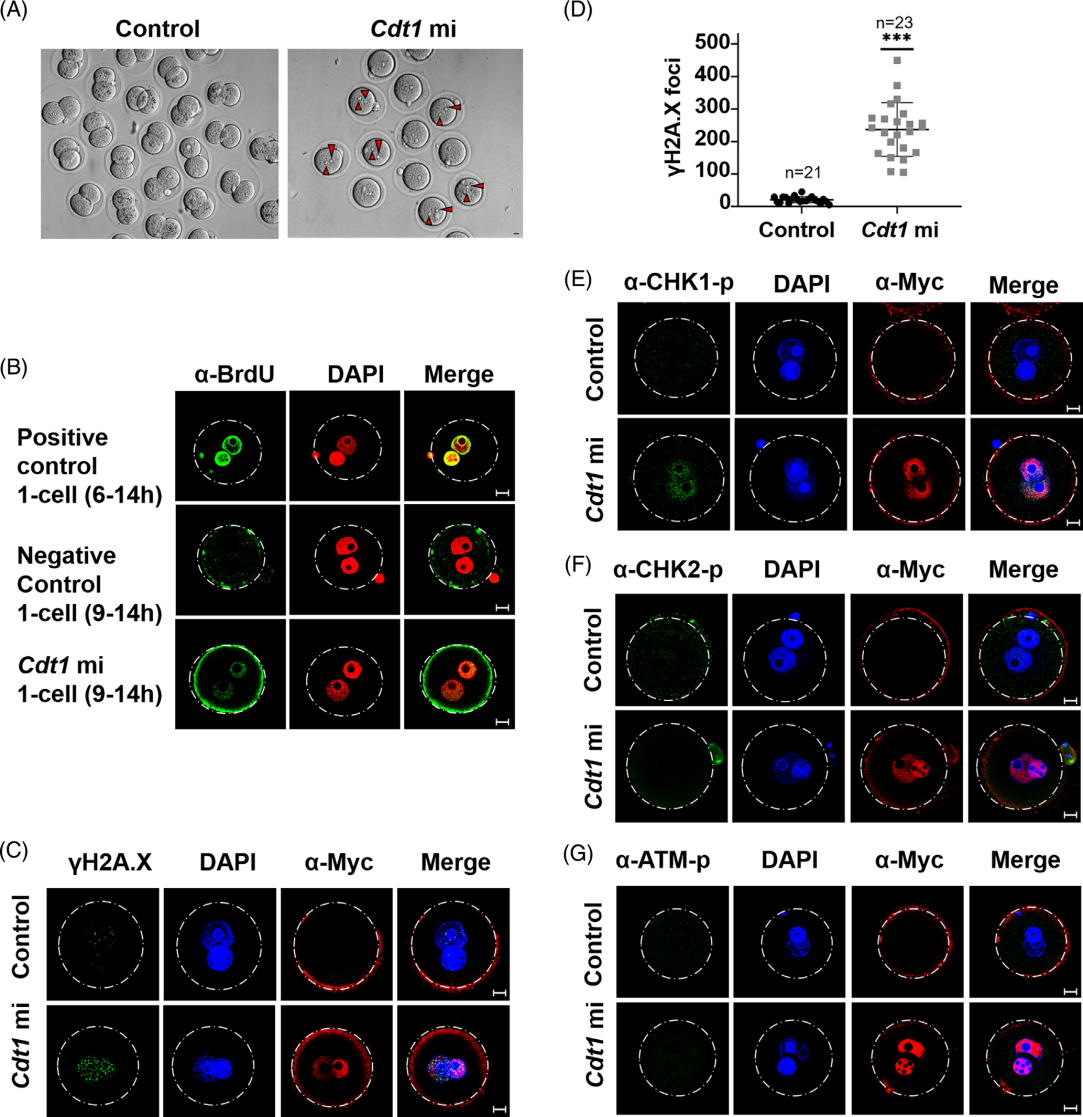
Exogenous expression of *Cdt1* leads to embryonic development arrest. (A) Fertilized eggs microinjected with *Cdt1* mRNA at IVF 9 h were arrested at 1 cell stage. (B) Confocal micrographs of BrdU labelling in embryos at IVF 14 h. 1‐cell (6–14 h) and 1‐cell (9–14 h) represented embryos transferred to KSOM medium supplemented with BrdU at 6–14 h and 9–14 h, respectively. (C) Representative immunofluorescence images of γH2A.X in embryos^exo‐Cdt1^. (D) Statistics of γH2A.X foci in zygotes microinjected *Myc‐Cdt1* mRNA and nuclease‐free water (***p < 0.001). (E) Representative immunofluorescence images of α‐CHK1 phosphorylation staining in embryos^exo‐Cdt1^. (F) Representative immunofluorescence images of phosphorylated CHK2 in embryos^exo‐Cdt1^. (G) Representative immunofluorescence images of phosphorylated ATM in embryos^exo‐Cdt1^. Scale bar: 10 μm

After fertilization, embryos need to amplify their genomes through DNA replication. Our previous study showed that depletion of geminin impairs early embryo development, probably by altering *Cdt1* expression and DNA re‐replication.^14^ Zygote expression profile of *Cdt1* showed decreased CDT1 in the G2 phase (Figure 1D). These aroused our interest in whether degradation of CDT1 was essential for mammalian embryo development. We injected *Myc*‐*Cdt1* mRNA at IVF 9 h, corresponding to the G2 phase and found that embryos were arrested at zygote stage (Figure 4A). Because CDT1 is an original factor of DNA replication, to detect whether embryonic development arrest was due to excessive DNA replication, we examined the BrdU signals in zygotes at IVF 9‐14 h, and found that CDT1 overexpression in the G2 phase caused new DNA synthesis (Figure 4B). Re‐replication is typically associated with activation of DNA damage response pathway.^9^, ^15^, ^16^ And abnormal DNA replication may lead to DNA damage. We did find that *Cdt1* mRNA microinjection caused severe DNA damage as shown by γH2A.X staining (Figure 4C). Furthermore, γH2A.X foci statistical analysis also showed that overexpression of CDT1 resulted in more serious DNA damage (Figure 4D).

Cellular responses to DNA damage are coordinated by DNA double‐strand breaks (DSBs) and single‐stranded DNA, which activate two distinct kinase signalling cascades: ATM–CHK2 and ATR–CHK1 pathways.^17^ To confirm the pathway that responds to DNA damage, we detected CHK1 and CHK2 phosphorylation by immunofluorescent staining. As shown in Figure 4E, there were stronger CHK1‐p signals compared to the control group, while we did not find any CHK2‐p (Figure 4F). Meanwhile, no ATM phosphorylation signals were detected (Figure 4G). Since CHK1 controls the timing of mitotic entry, it may be that activation of CHK1 prevents mitotic G2/M transition.

### 3.5 MCM2 is dispensable for oocytes meiotic maturation and embryonic development

**FIGURE 5:**
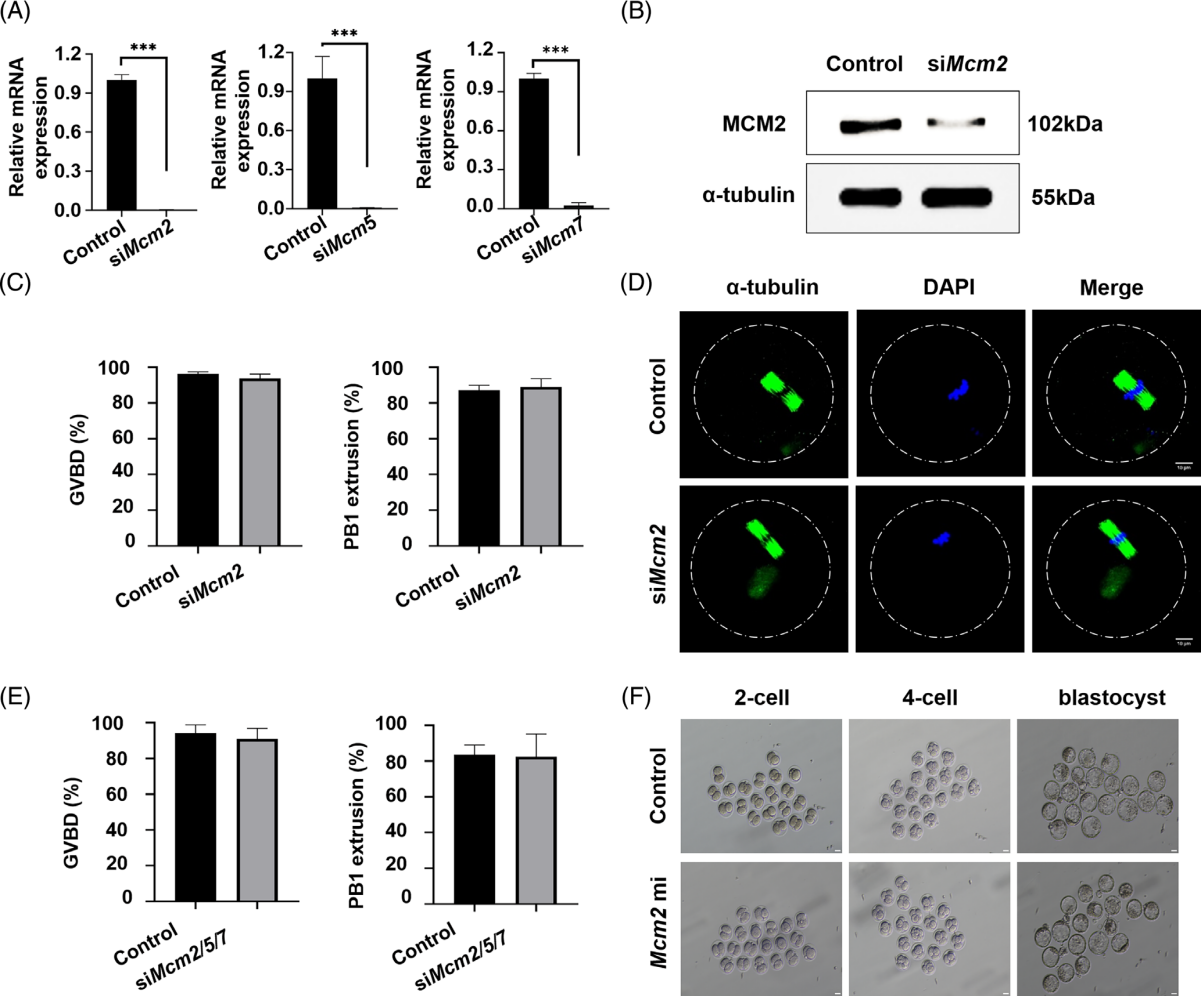
MCM2 is dispensable for oocyte meiotic maturation and embryonic development. (A) Relative mRNA levels of *Mcm2*, *Mcm5* and *Mcm7* in the oocytes injected with specific siRNA respectively or control siRNA. The oocytes were arrested at GV stages for 24 h (***p < 0.001). (B) Western blot of MCM2 in GV oocytes injected with *Mcm2* siRNA and control siRNA. The molecular mass of MCM2 is 102 kDa and that of α‐tubulin is 55 kDa. Each sample contained 120 oocytes. (C) The GVBD and PBE rates of oocytes injected with *Mcm2* siRNA or control siRNA. A total of 131 oocytes in control group and 82 oocytes in siRNA group were used from three replicates. (D) The spindles of MII of oocytes injected with *Mcm2* siRNA or control siRNA. Oocytes were cultured for 24 h in M2 medium with milrinone, then washed and moved to milrinone free M2 medium followed by immunofluorescent staining. (E) The GVBD and PBE rates of oocytes injected with *Mcm2*, *Mcm5* and *Mcm7* siRNA or control siRNA. A total of 102 oocytes in control group and 78 oocytes in siRNA group were used from three replicates. (F) Embryos microinjected with *Mcm2* mRNA developed to blastocysts. Scale bar: 10 μm

Given that CDT1, CDC6 and MCM complex jointly regulate replication initiation, and that CDC6 was involved in regulating both G2/M transition and metaphase‐to‐anaphase transition during oocyte meiosis as we previously reported.^13^ So we supposed that MCM complex may play other roles in addition to regulating DNA replication initiation. We knocked down *Mcm2*, *Mcm5* and *Mcm7* by microinjecting siRNA respectively. *Mcm2*, *Mcm5* and *Mcm7* mRNA levels were measured by qRT‐PCR. Compared with control group, *Mcm2*, *Mcm5* and *Mcm7* mRNA were notably reduced (Figure 5A). Likewise, the protein abundance of MCM2 was also decreased in *Mcm2* knock‐down group (Figure 5B).

When *Mcm2* expression was depleted, the rates of GVBD and PBE were similar between control group and knockdown group (Figure 5C). Meanwhile, the spindle morphology was also normal (Figure 5D). To exclude the possibility of the redundancy of different MCM subunits, we injected three MCM subunits specific siRNA in combination, but still did not find any abnormality in knockdown group (Figure 5E). Furthermore, due to exogenous expression of *Cdt1* in G2 led to zygote stage arrest, we exogenously expressed *Mcm2* in embryos to explore the function of *Mcm2*, however, most embryos developed to blastocysts (Figure 5F).

## 4 DISCUSSION

It has been showed that DNA replication is precisely regulated during cell mitosis, and replication factors work together to regulate the process. ORC directly binds DNA, which is essential for the formation of pre‐RC. CDT1 is recruited to DNA through interaction with ORC.^18^, ^19^ MCM complex is a hexameric AAA+ ATPase, which is a motor of DNA helicase recruited to pre‐RC through combining with CDT1.^20^, ^21^

CDT1 plays multifunction during cellular mitosis. Previous report suggests that CDT1 localizes to kinetochores through interaction with HEC1, and its depletion leads to cell arrest at prometaphase during mitosis.^12^ Here, we hypothesize that CDT1 may play a role in meiotic cell cycle progression. We showed that CDT1 was localized at nucleus in the GV oocytes. When depleted CDT1 in GV oocytes by specific siRNA, the GVBD and PBE rates showed no obvious difference compared to the control group, and M II spindle morphology and chromosome alignment were not disrupted as well, suggesting that unlike in mitosis, CDT1 is not involved in regulating meiotic progression.

**FIGURE 6:**
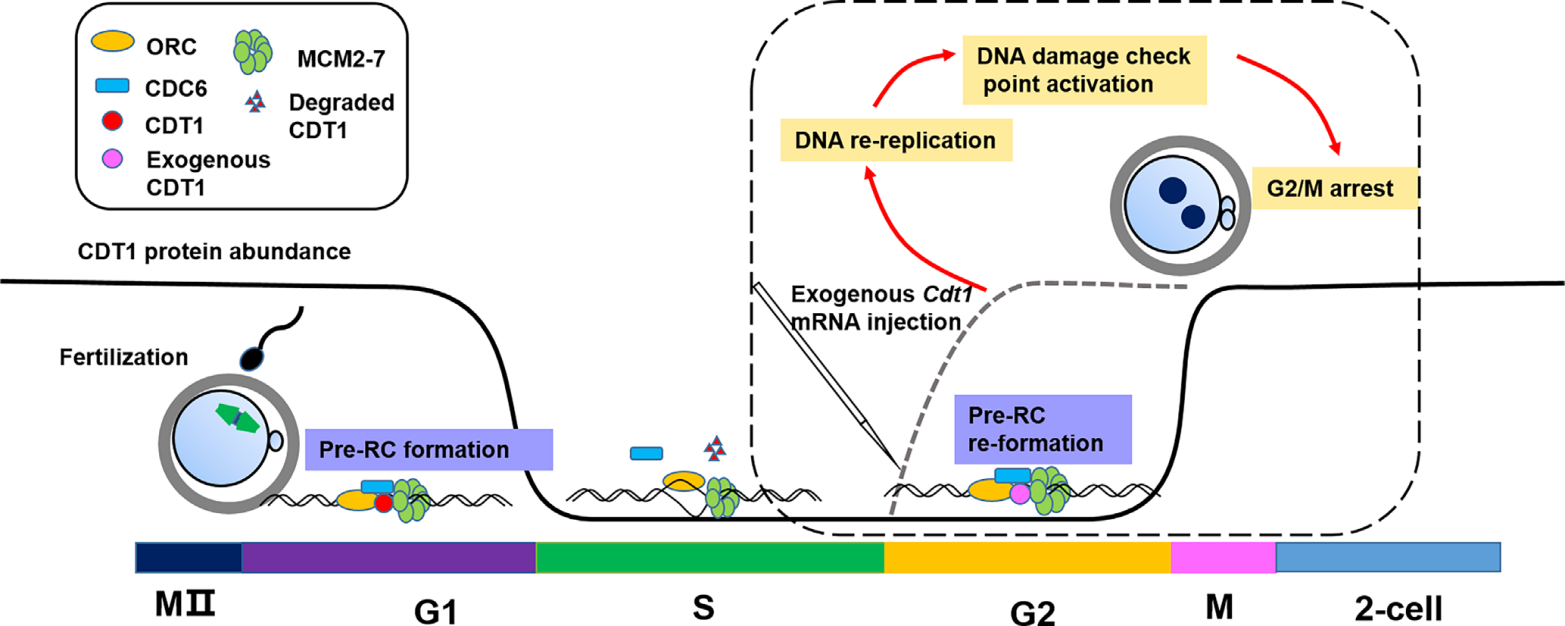
Schematic figure showing possible mechanism of G2/M arrest in zygotes injected with exogenous *Cdt1* mRNA: Pre‐RC is formed in G1 phase during which CDT1 is localized in nucleus. When zygote enters S phase, CDT1 is degraded and stays at a low level in G2 phase. However, the expression of exogenous *Cdt1* mRNA in G2 phase of fertilized egg, mimicking events in which CDT1 degradation is disrupted, pre‐RC reassembles and leads to DNA re‐replication, and thus DNA damage check point activation, which results in embryo arrest at G2/M phase.

Previous study shows that the protein abundance of CDT1 is high at G1 and G2 stages in mitosis, and it is degraded at S phase in proliferating cells.^22^ During S phase, CDT1 is ubiquitinated by two independent E3 ubiquitin ligase complexes, CRL1^Skp2^ ^4^ and CRL4^Cdt2^,^5^, ^6^ or is blocked by geminin from binding to MCM complexes and CDC6.^23^, ^24^ In our study, we found that CDT1 was localized at cell nuclei of embryo in G1 phase, degraded in S phase, and did not re‐accumulate at nucleus in G2 phase. To explore the function of CDT1 absence in G2 phase of mammalian zygotes, we exogenously expressed *Cdt1* in G2 phase, and the results showed that embryos initiated DNA re‐replication in G2 phase. In mammalian cells, overexpression of *Cdt1* can induce ATM/ATR‐dependent checkpoint activation.^9^ In this study, we detected increased γH2A.X and CHK1 phosphorylation signals, indicating the accumulation of DSB and thus DNA damage repaired pathway activation. CHK1 is G2 checkpoint kinase, and DNA re‐replication induced DSB formation and check points pathway activation that blocks further cell cycle progression. Coincidently, our results show that the overexpression of *Cdt1* induces embryonic development arrested at zygote stages (Figure 6).

Recently, Palmerola *et al*. report that one‐cell embryos showed replication delay to G2 phase in human embryos.^25^ In our study, exogenous expression of *Cdt1* resulted in DNA re‐replication, which may be due to pre‐RC re‐formation in open chromatin regions. In comparison to human embryos, mouse embryos show a lower frequency of segregation errors when interference with aphidicolin in G2 phase, and this may be due to a lower number of un‐replicated sites in mice.^25^ What causes this difference? Confocal micrographs revealed that CDT1 is absent in G2 nucleus of one‐cell embryo, which may partly explain this phenomenon. The GV oocytes are arrested at prophase of meiosis I, and we also hypothesize the possibility that exogenous expression of *Cdt1* may induce DNA re‐replication，but our results showed that there were no BrdU labelling signals. The different situations between zygotes and oocytes may be due to the more condensed chromosomes of oocytes and the replication delay of zygotes.

MCM complex consists of six distinct subunits (MCM2‐7). MCM2, a subunit of MCM complex, is recruited to DNA by CDT1. Since exogenous expression of *Cdt1* in G2 phase results in DNA re‐replication in zygote, we decided to determine whether MCM2 had similar DNA replication regulatory function like CDT1. MCM2 was localized at nucleus from G1 to G2 stages, and embryos with exogenously expressed MCM2 could develop to blastocysts. We further investigated whether MCM2 had potential functions in meiotic oocytes. But exogenous expression or depletion of MCM2 did not affect GVBD and PBE rates.

Both CDT1 and MCM2 are involved in regulating DNA replication as subunits of pre‐RC, however, the protein abundance of CDT1 changes periodically, whereas MCM2 persists throughout the cell cycle. We found that overexpression of *Cdt1* leads to embryos arrest at zygotes stages, but the development with overexpressed MCM2 embryos is not affected, consistent with the expression patterns of *Cdt1* and *Mcm2*, revealing that CDT1 mainly regulates DNA replication as a regulator, while MCM2 is only involved in DNA replication as an ATPase. CDC6 has been shown to be involved in regulating meiosis in addition to being a subunit of PRC.^13^ and CDT1 is involved in regulating kinetochore–microtubule attachments in mitosis.^12^ However, in the present study, CDT1 and MCM2 are not required for oocytes meiotic maturation.

In summary, our study reveals that CDT1 but not MCM2 is the major regulator of DNA replication in 1‐cell embryo, it is important to appropriately regular CDT1 protein abundance to avoid DNA re‐replication in G2 phase. Furthermore, CDT1 and MCM2 are not involved in regulating oocytes meiotic maturation apart from their role in forming pre‐RC.

## AUTHOR CONTRIBUTIONS

Chao Li and Tie‐Gang Meng performed main experiments. Yong‐Peng Tan collected samples. Chao Li performed data analysis and manuscript writing. Xue‐Shan Ma and Zhen‐Bo Wang provided revisions. Tie‐Gang Meng and Qing‐Yuan Sun designed the project, reviewed and edited manuscript.

## CONFLICT OF INTEREST

The authors declare no competing interests.

## Data Availability

The data used to support the findings of this study are available from the corresponding author upon request.
